# Interplay of m6A RNA methylation and gut microbiota in modulating gut injury

**DOI:** 10.1080/19490976.2025.2467213

**Published:** 2025-02-17

**Authors:** Haixia Wang, Juanjuan Han, Xin-An Zhang

**Affiliations:** College of Exercise and Health, Shenyang Sport University, Shenyang, China

**Keywords:** N6-methyladenosine, microbiota, intestinal flora, gut injury, intestinal homeostasis

## Abstract

The gut microbiota undergoes continuous variations among individuals and across their lifespan, shaped by diverse factors encompassing diet, age, lifestyle choices, medication intake, and disease states. These microbial inhabitants play a pivotal role in orchestrating physiological metabolic pathways through the production of metabolites like bile acids, choline, short-chain fatty acids, and neurotransmitters, thereby establishing a dynamic “gut-organ axis” with the host. The intricate interplay between the gut microbiota and the host is indispensable for gut health, and RNA N6-methyladenosine modification, a pivotal epigenetic mark on RNA, emerges as a key player in this process. M6A modification, the most prevalent internal modification of eukaryotic RNA, has garnered significant attention in the realm of RNA epigenetics. Recent findings underscore its potential to influence gut microbiota diversity and intestinal barrier function by modulating host gene expression patterns. Conversely, the gut microbiota, through its impact on the epigenetic landscape of host cells, may indirectly regulate the recruitment and activity of RNA m6A-modifying enzymes. This review endeavors to delve into the biological functions of m6A modification and its consequences on intestinal injury and disease pathogenesis, elucidating the partial possible mechanisms by which the gut microbiota and its metabolites maintain host intestinal health and homeostasis. Furthermore, it also explores the intricate crosstalk between them in intestinal injury, offering a novel perspective that deepens our understanding of the mechanisms underlying intestinal diseases.

## 1. Introduction

M6A, also known as N6-methyladenosine, constitutes over 80% of RNA methylation modifications, with approximately 1/3 to 1/2 of human and mouse messenger RNA (mRNA) undergoing m6A modification.^1,2^ To date, over 160 different types of RNA chemical modifications have been identified.^3,4^ Among them, m6A is the most abundant and distinctive modification in both coding and non-coding RNA (ncRNA). It is mainly enriched near the stop codon, 3’ untranslated regions (3’ UTRs), precursor mRNA, and the inner and outer exons of mature mRNA.^5–7^ These regions are usually closely related to processes such as mRNA decay, splicing, and translation.^8–11^ Research has firmly established that this modification exhibits sequence conservation, primarily occurring in G(m6A) C (70%) or A(m6A) C (30%) motifs.^12,13^ Since its discovery in mammalian mRNA in the 1970s, m6A research has emerged as a frontier in science.^14,15^ Recent technological advancements, particularly high-throughput sequencing, have deepened our understanding of m6A’s pivotal role in gene expression regulation, cellular function, disease onset, and plant growth.^16–19^

The gut microbiota, a vast microbial ecosystem within humans, significantly influences host physiology and pathology, encompassing metabolism, intestinal homeostasis, and immune system development.^20,21^ Recent studies suggest that disruptions in epigenetic transcriptome mechanisms, including m6A modification dynamics, contribute to intestinal diseases.^22^ The gut microbiota, via metabolites, signaling molecules, and direct cell-cell interactions, modulates the activity and expression of RNA-modifying enzymes in host cells, thereby regulating m6A modification levels.^23,24^ This regulation exhibits tissue specificity and may vary with disease status. However, the precise mechanisms underlying gut microbiota’s influence on host RNA m6A modification and their interactions remain unclear. This review sheds light on the roles of epigenetic m6A modifications and gut microbiota in intestinal diseases, emphasizing their interplay in intestinal injury and maintenance of intestinal structure-function homeostasis.

## 2. M6A modification and gut injury

**Figure 1.**
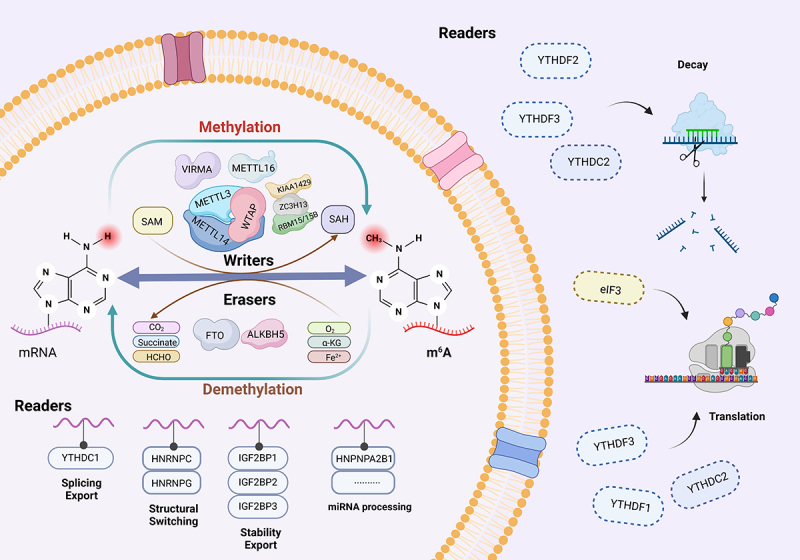
Composition and mechanism of m6A RNA methylation. M6A methylation is a dynamic and reversible process, where m6A modifications are added by the “writer” (m6A methyltransferase), removed by the “eraser” (demethylase), and recognized by the “reader” (m6A binding protein) to mediate their function. M6A methylation plays an important role in multiple aspects of RNA metabolism, including RNA stability, splicing, enucleation, localization, and translation.

The discovery of M6A in mRNA in 1974 heralded a groundbreaking revelation in the realm of eukaryotic RNA modifications. This ubiquitous modification, enriched in distinct regions of mRNA, exhibits remarkable conservation and sequence specificity, profoundly influencing RNA expression and functionality, as well as participating in the intricate regulation of ncRNA. The intricate m6A modification cycle encompasses three key steps: “writing” orchestrated by methyltransferases like methyltransferase-like 3/14/16 (METTL3/14/16), “erasing” facilitated by demethylases such as fat mass and obesity-associated protein (FTO) and alkB homologue 5 (ALKBH5), and “reading” regulated by reader proteins belonging to the YT521-B homology (YTH) family. This dynamic and reversible process meticulously modulates RNA stability, splicing, and various other aspects^25^ (Figure 1). Recent years have witnessed a surge in research emphasizing the pivotal role of m6A methylation modification in intestinal diseases, encompassing stem cell pluripotency, cell differentiation, proliferation, metastasis, anti-tumor immunity, and chemotherapy resistance.^26,27^

### 2.1. M6a writers

M6A writers, enzyme complexes, catalyze m6A modification in RNA. Key players METTL3, METTL14, and Wilms tumor 1-associated protein (WTAP) regulate intestinal homeostasis.^28^ METTL3 notably impacts disease progression by modulating inflammation, apoptosis, and autophagy. In inflammatory bowel disease (IBD) patients, METTL3 levels surge,^29^ correlated with elevated m6A modification and heightened colonic inflammation via TLR4/NF-κB pathway activation in macrophages.^30^ This triggers NF-κB signaling, boosting epithelial apoptosis, proinflammatory cytokine expression, and p65 phosphorylation, exacerbating IBD pathology.^31^ Conversely, METTL3 deletion dampens inflammation by inhibiting CD4+ T helper 1 (Th1) differentiation via YTHDF3-mediated phosphoglycolate phosphatase (PGP) upregulation.^32^ It is crucial that METTL3-mediated m6A also upregulates circPRKAR1B, intensifying NLRP3 inflammasome-mediated pyroptosis via autophagy damage in Crohn’s colitis.^33^ Furthermore, METTL3 enhances SOX2 expression, modulating m6A-CRB3-Hippo, mTORC1, and JAK1/STAT3 signaling, thereby promoting colorectal cancer (CRC) progression.^34–37^ METTL14 is pivotal for preserving intestinal epithelial homeostasis, with its absence causing mucosal barrier malfunction and shortened lifespan in mice. Typically, METTL14 hinders colonic cell death via the NF-κB-mediated anti-apoptotic pathway.^38^ However, METTL14 deficiency disrupts this balance, diminishing GSDMC levels and initiating the proapoptotic cascade involving PUMA, Caspase 3, and 9, while disrupting colonic organoid growth and differentiation.^39^ Furthermore, this deficiency intensifies inflammation in Caco-2 cells and impedes autophagy.^40^ In CRC, METTL14 specifically promotes the maturation of miR-6769b and miR-499a precursors via the m6A-YTHDF2 pathway, suppressing SLC2A3 and PGAM1 expression, thus cutting off energy supply in p53-WT CRC cells.^41^ Additionally, METTL14 may halt CRC progression by downregulating oncogenic lncRNA XIST and inhibiting the SOX4-mediated PI3K/Akt pathway.^42,43^ While WTAP lacks direct methyltransferase activity, it stabilizes the m6A methylation complex,^44^ crucial for colitis prevention by activating intestinal RORγT to modulate T cell function.^45^ Like METTL3, WTAP mRNA expression is upregulated in ulcerative colitis (UC) patients, serving as a sensitive diagnostic marker.^29^ Overexpression of WTAP promotes cell proliferation, migration, invasion, and angiogenesis in CRC, while knockout inhibits these malignant behaviors.^46^ Mechanistically, WTAP upregulates VEGFA and activates the MAPK pathway, enhancing angiogenesis.^47^ It may also dampen antitumor T cell responses and foster CRC cell proliferation by modifying PD-L1 and FLNA 3′UTRs.^48,49^ Notably, WTAP can be targeted by ARRB2 and carbonic anhydrase IV (CA4) for degradation, thereby promoting CRC cell growth and migration.^50,51^

### 2.2. M6a erasers

FTO and ALKBH5, m6A demethylases, play pivotal roles in intestinal injury by modulating ferroptosis and inflammation. FTO upregulation in CRC elicits a colitis-like phenotype.^52–54^ Studies confirm that FTO, through YTHDF2-dependent m6A modification, degrades the apoptosis gene siva1 mRNA, downregulates solute carrier family 7 member 11 (SLC7A11) and glutathione peroxidase 4 (GPX4), and thereby fosters CRC tumorigenicity and ferroptosis.^53,55^ Specifically, FTO demethylates GPX4 mRNA at site 193, enabling YTHDF2 to recognize and degrade it, promoting CRC cell iron death.^56^ Additionally, FTO demethylates G6PD/PARP1, influencing DNA damage and repair, and advancing CRC progression and chemoresistance.^57^ It also enhances CRC growth, metastasis, and angiogenesis via the Wnt/β-catenin pathway, mediated by ZNF687.^58^ However, it is worth noting that FTO downregulation leads to increased sphingosine-1-phosphate (S1P) accumulation in intestinal epithelial cells by reducing the mRNA stability of CerS6, a gene encoding ceramide synthase, and aggravates UC through m6A dependent mechanisms.^59^ On the other hand, hypoxia-induced downregulation of FTO paradoxically enhances CRC growth and metastasis, despite FTO’s tumor-suppressing role by inhibiting metastasis-associated protein 1 (MTA1) expression. Interestingly, this suppression primarily manifests through reduced FTO protein levels, not mRNA transcription, underscoring intricate transcriptional regulatory dynamics.^60^ Similar to FTO, high expression of ALKBH5 predicts poor CRC prognosis, yet its reduction ameliorates experimental colitis.^52,61^ In particular, ALKBH5 demethylates AXIN2 mRNA, freeing it from IGF2BP1 and activating Wnt/β-catenin, which induces Dickkopf-related protein 1 (DKK1) to recruit myeloid derived suppressor cells (MDSCs), fostering CRC immunosuppression.^61^ Functionally, ALKBH5-mediated demethylation also activates RAB5A, thwarting YTHDF2-mediated degradation, thereby bolstering CRC cell proliferation, migration, and invasion.^62^ In addition, it promotes CRC by upregulating CPT1A and fatty acid metabolism,^63^ yet paradoxically reduces CRC tumorigenesis and colitis risk. ALKBH5 reportedly safeguards mice from colitis tumors,^64^ its expression declines in CRC mouse models. However, ALKBH5 overexpression inhibits colon cancer and CRC cell proliferation, migration, and invasion.^65^ Mechanistically, ALKBH5 can destabilize PHF20 mRNA by demethylating its 3‘UTR, hindering CRC, and boost CD8 T-cell infiltration in CRC microenvironments by inhibiting NF-κB-CCL5 signaling, mitigating malignancy.^66,67^ Notably, high-fat diets downregulate FTO and ALKBH5 in CRC patients, reducing Hexokinase 2 (HK2) via m6A-IGF2BP2. This modulates METTL3/14 and activates FOXO pathways, fueling CRC progression.^68^ In summary, environmental influences may have a significant impact on how m6A regulatory factors work in illnesses.

### 2.3. M6a readers

The m6A reader recognizes and binds to RNA sequences modified with m6A, key players being YTHDF1/2/3, YTHDC1/2, and IGF2BP1/2/3, which significantly impact intestinal health. Specifically, intestinal damage is helped by YTHDF1/3, but YTHDF2 has two effects. YTHDF1 fosters intestinal injury, tumor growth, and metastasis by boosting ARHGEF2 translation and CXCL1/CXCR2 activation via NF-κB p65, hindering T-cell infiltration and aiding CRC progression.^69–71^ This process is also closely related to the activation of the Wnt/β-catenin pathway.^72^ In contrast, the mechanism of action of YTHDF2 is more complex. Targeting down-regulation of YTHDF2 increases the stability of m6A-modified GSK3β mRNA and inhibits the Wnt/β-catenin/Cyclin D1 pathway, which in turn prevents CRC cell proliferation.^73^ But by identifying its m6A mutation, YTHDF2 may also control the degradation of circYAP1, which prevents CRC cells from proliferating, invading, migrating, and escaping the immune system.^74^ The diverse kinds of m6A modified RNAs that YTHDF2 targets in various illness contexts and cellular settings may be the cause of this dual impact. YTHDF3 is significantly expressed in CRC. It primarily recognizes the 5’ UTR of m6A methylated RNA, enhances the stability of the eukaryotic translation initiation factor 3 subunit A (eIF3A) complex through eukaryotic translation initiation factor 2 alpha kinase 2 (eIF2AK2), and promotes target gene translation efficiency.^75^ Additionally, YAP-mediated CRC cell invasion and proliferation were reduced when YTHDF3 was knocked down.^76^ YTHDC1/2 presence bolsters intestinal health. In IBD colonic macrophages, YTHDC1 expression declines significantly, inhibiting m6A-mediated upregulation of Ras homolog family member H (RHOH) and NME nucleoside diphosphate kinase 1 (NME1), thus exacerbating inflammation and disrupting colonic barrier integrity.^77^ Like YTHDC1, YTHDC2 also suppresses tumors.^78^ YTHDC2 downregulation decreases m6A recognition of LIM kinase 1 (LIMK1) mRNA, stabilizing LIMK1 and inducing eIF2α phosphorylation, ER stress, and ultimately 5-Fluorouracil (5-FU) resistance in colorectal cancer.^79^ Controversial is the function of the IGF2BP protein family as m6A readers in intestinal homeostasis. High expression of IGF2BP1 is associated with poor prognosis in CRC, as it alters the transcriptional profile of CRC mRNA from the extracellular vesicle (EV) pathway.^80^ IGF2BP2 modulates iron metabolism and CRC progression by METTL4-mediated TFRC mRNA methylation.^81^ Yet, IGF2BP1 safeguards intestinal barrier integrity by regulating occludin.^82^ IGF2BP2 enhances GPX4 expression through m6A modification, inhibits ferroptosis, and thus weakens UC progression.^83^ In addition, IGF2BP3 enhances the occurrence and progression of CRC tumors in vitro and in vivo.^84^ Mechanistically, IGF2BP3 promotes the stability and translation of epidermal growth factor receptor (EGFR) mRNA, and collaborates with METTL14 in m6A dependent manner.^85^ In conclusion, m6A regulatory factors exhibit remarkably diverse functionalities across varying disease contexts and cellular milieus, offering fresh insights and posing intricate challenges for the precise therapeutic targeting of intestinal disorders.

**Figure 2.**
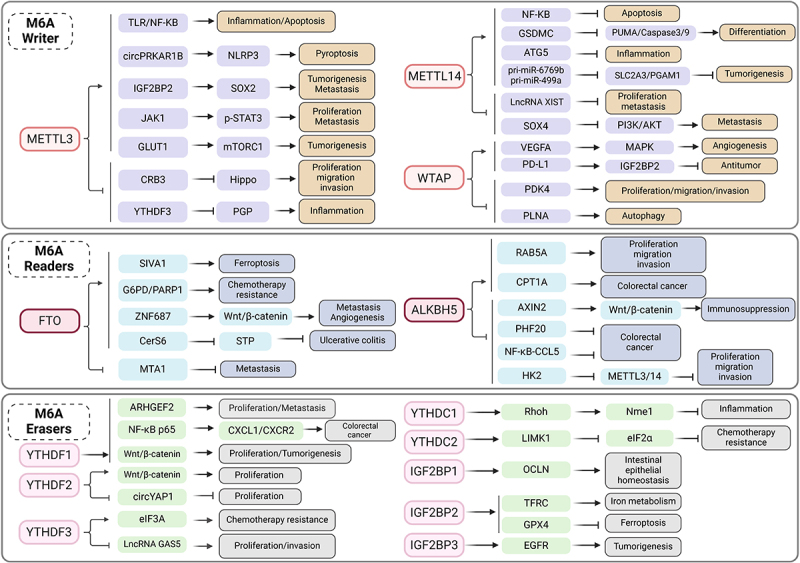
Mechanism of M6A regulating intestinal injury. M6a writers (METTL3, METTL14, and WTAP), M6a readers (FTO and ALKBH5) and M6a erasers (YTHDF1/2/3, YTHDC1/2, and IGF2BP1/2/3) can affect intestinal inflammatory reaction, apoptosis, differentiation, autophagy, proliferation, migration, invasion, ferroptosis, etc. It also regulates immunosuppression, chemotherapy resistance and tumorigenesis and development, thus affecting intestinal damage and causing intestinal related diseases, such as ulcerative colitis and colorectal cancer.

In summary, the intricate interplay between RNA m6A modification and intestinal injury underscores a profound connection (Figure 2). Thorough exploration of the underlying regulatory mechanisms not only illuminates the intricate pathogenesis of intestinal diseases but also harbors the potential to usher in novel preventive and therapeutic strategies, offering promising avenues for targeting and managing related disorders.

## 3. Gut microbiota and gut injury

**Figure 3.**
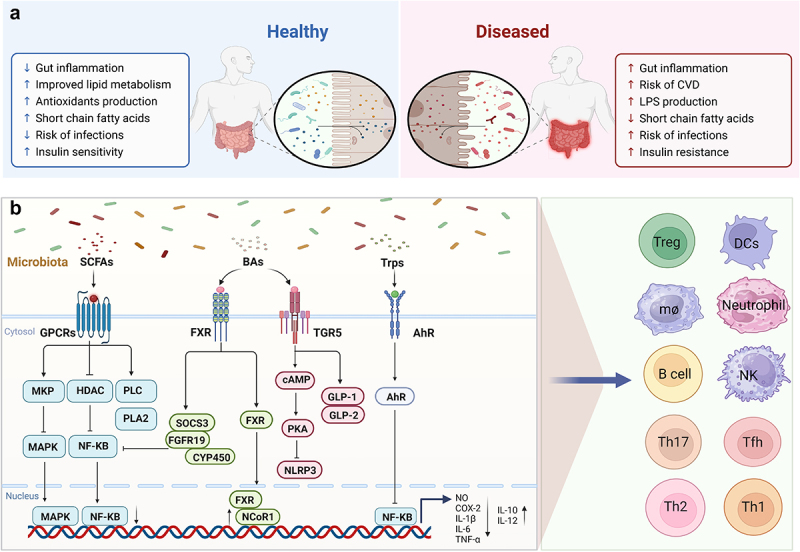
The mechanism by which gut microbiota affects gut health. (a) In physiological states, the gut microbiota fosters intestinal homeostasis, mitigating inflammation and disease incidence. Conversely, under pathological conditions, the delicate balance of the gut microbiota is perturbed, compromising the host’s physiological functions and predisposing to disease development. (b) The gut microbiota exerts its influence on host health via metabolic and signaling interactions mediated by metabolites like short-chain fatty acids, bile acids, and tryptophan. This intricate communication precisely modulates the function and activity of immune cells, encompassing Tregs, dendritic cells, macrophages, diverse T lymphocyte subsets (Th1, Th2, Th17), and B lymphocytes, thereby preserving intestinal homeostasis and systemic immune equilibrium.

The human gut microbiota is a complex ecosystem, and the “microbiome,” or collection of its genes, has a significant impact on physiological processes.^86^ The two most common phyla among the many kinds of microbes are Phylum Firmicutes and Bacteroidetes. By secreting metabolites like bile acids (BAs) and short chain fatty acids (SCFAs), microorganisms control immunological responses, metabolic activities, and intestinal barrier function, preserving intestinal homeostasis and systemic immune balance. Together with epithelial tissue, they use metabolism and signal transmission to protect the host from outside invasion and to advance host health (Figure 3). The microbiome also regulates the hypoxic environment of the intestine, which is crucial for nutrient absorption by intestinal mucosal cells, intestinal barrier function, and immune response.^87,88^ The development of the immune system, intestinal homeostasis, and host metabolism are all significantly impacted by the gut microbiota and its metabolites.^89–91^ The gut microbiota significantly impacts gut health and homeostasis, contributing to biological processes underlying various diseases.^92,93^ Key intestinal microbes like Fusobacterium nucleatum (F. nucleatum), Escherichia coli (E. coli), and Bacteroides fragilis (B. fragilis) are pivotal in intestinal injury, disrupting barriers, instigating inflammation, and fostering intestinal tumors. Moreover, microbiota imbalance can spur overgrowth or infection of other microorganisms, intensifying intestinal damage.

### 3.1. Fusobacterium nucleatum

F. nucleatum promotes tumor growth, invasion, and metastasis, while disrupting immune homeostasis via downstream signaling activation. Research confirms its abundance in CRC and adenoma patients, attributing to its pro-inflammatory and tumor-promoting effects.^94,95^ By invading intestinal epithelial cells, F. nucleatum binds to DHX15 receptor, to activate ERK-STAT3 signaling, thereby fostering intestinal tumor growth.^96^ In addition, CRC cells infected with F. nucleatum upregulate matrix metalloproteinase 7 (MMP7) by activating the MAPK (JNK)-AP1 pathway, accelerating cell migration.^97^ Intriguingly, F. nucleatum directly engages with docking molecules on CRC stem cells, enhancing adhesion, initiating SHP-2 signal transduction and tyrosine phosphorylation cascades, thereby contributing to CRC development.^98^ Similarly, F. nucleatum upregulates IRF5 via the lncRNA ENO1-IT1/miR-22-3p pathway, intensifying the inflammatory progression of necrotizing enterocolitis (NEC).^99^ F. nucleatum not only fosters intestinal tumor growth but also perturbs immune balance. It recruits tumor associated macrophages (TAMs) and dendritic cells (DCs), depletes T cells, reduces FoxP3 regulatory T cell enrichment, and dampens NK cell activity, suppressing anti-tumor immunity and exacerbating pro-inflammatory CRC mechanisms.^100–102^ On the one hand, it enhances M2 macrophage polarization (ARG-1, IL-10, MRC1, TGF-β) and CCL20 expression via NF-κB/miR-1322, facilitating CRC metastasis.^103^ On the other hand, it activates ALPK1-mediated NF-κB, upregulating ICAM1 to promote CRC adhesion, extravasation, and metastasis to the endothelium.^104^

F. nucleatum not only directly invades the intestinal mucosa, but also accelerates intestinal epithelial cell barrier damage through its derived extracellular vesicles (EVs), exosomes, and outer membrane vesicles (OMVs). F. nucleatum shed EVs (FnEVs) spur macrophage release of pro-inflammatory cytokines, activate RIPK1/RIPK3-mediated apoptosis, hinder intestinal epithelial cells (IECs) renewal, and accelerate UC progression.^105^ F. nucleatum-EVs also mediates autophagy pathway to exacerbate experimental colitis and epithelial barrier damage. It downregulates miR-574-5p to inhibit CARD3, upregulates LC3II and inflammatory factors (IL-1 β, IL-6, and TNF - α), and reduces ZO-1 and occludin.^106^ F. nucleatum infected IECs derived exosomes (F. nucleatum-Exo) activate the ATM/ATR/p53 pathway through the miR-129-2-3p/TIMELESS axis, disrupting the intestinal mucosal barrier, promoting cellular aging, and exacerbating experimental colitis.^107^ Moreover, F. nucleatum also adheres to intestinal mucus and secretes virulence factor OMVs. OMVs can activate innate immune responses, such as epithelial TLR4, thereby activating RK, CREB, and NF-κB related pathways and driving pro-inflammatory cytokine responses.^108^

F. nucleatum poses a significant threat to intestinal health due to its metabolic byproducts, including formate, SCFAs, hydrogen sulfide (H_2_S), and ADP heptose. Formate promotes the expansion of Th17 T cells in CRC. Supplementing with formate can increase the number of CD4IL-17CORγT cells in mice and enhance cancer stem cell (CSCs) stemness through AhR signaling, thereby improving cancer cell invasiveness.^109^ Similarly, SCFAs produced also induce Th17 cells to affect intestinal immunity.^110^ H_2_S and ADP heptose exacerbate the intestinal inflammatory response. H_2_S upregulates the expression of inflammatory factors (VEGF, COX-2 and IL-6) and autophagy related genes DRAM1, NBR1, and MAP1LC3A in mouse colon tissue.^111^ Importantly, H_2_S disrupts the normal gut microbiota, reduces α diversity in mouse gut microbiota, and alters β diversity.^111^ The ADP heptose activates the intestinal epithelial NF- κB pathway through ALPK1/TIFA/TRAF6 signaling, upregulating IL-8, anti-apoptotic factors (BIRC3 and TNFAIP3), and cell adhesion factor ICAM1. Simultaneously activating the autophagy pathway, but independently of the ALPK1/TIFA signaling.^112^ F. nucleatum treatment not only modulates metabolite levels but also regulates gut microbiota dysbiosis, notably reducing butyrate-producing bacteria, thereby hindering butyric acid synthesis.^113^ It also enriched pathogens linked to CRC development, including intestinal oncogenes, PPAR signaling pathway of cyclic adenosine monophosphate, and recruited eight taxonomic groups of Proteobacteria to change mucosal microflora.^114^

In summary, F. nucleatum plays multiple key roles in intestinal homeostasis and the occurrence and development of tumors. These studies have enriched our understanding of the pathogenesis of F. nucleatum and provided new perspectives for the development of intestinal injury and diseases.

### 3.2. Escherichia coli

E. coli, a ubiquitous bacterium in the gastrointestinal tract of mammals, possesses the ability to elicit the release of inflammatory factors from intestinal mucosal cells. Crohn’s disease (CD) patients have an increase in Escherichia coli in the small intestinal mucosa, and its specific strains can stimulate CD4+T cell responses and intestinal inflammation.^115^ Similarly, the ST129 and ST375 strains of E. coli have been shown to induce robust expression of various pro-inflammatory cytokines, including GM-CSF, IL-6, IFN-γ, IL-1β, IL-17A, and IL-33, in the cecum of mice.^116^ E. coli pathogenic bacteria aggravate intestinal harm through virulence factor production. For example, the ZB-1 strain secretes yersiniabactin (Ybt), activating the NLRP3 inflammasome. This cascade prompts cleavage of pro-caspase-1 into caspase-1 and GSDMD into GSDMD-N, ultimately fostering apoptosis and inflammation in intestinal epithelial cells.^117^ Pathogenic bacterium p19A adheres to the cecal mucosa of mice, causing atrophy and inflammation of cecal tissue by producing alpha-hemolysin (HlyA), exacerbating the course of UC.^118^ The HlyA carried by B2 type Escherichia coli can infect colon cancer cell line Caco-2, altering the ratio of PIP2 to PIP3 in the membrane, leading to changes in cell polarity and disruption of cell connections, ultimately causing intestinal epithelial cell shedding and death.^119^ Furthermore, an Escherichia coli O4 strain, via HlyA, decreases transepithelial resistance (R(t)) in rat colon and induces focal barrier leakage in HT-29/B6 cells.^120^ It is worth noting that enteroaggregative E. coli (EAEC), a noninvasive pathogen, adheres to intestinal mucosa, releasing toxins that ignite inflammation, resulting in secretory diarrhea and mucosal harm.^121^ Conversely, adherent-invasive E coli (AIEC), a truly invasive bacterium, colonizes the intestinal mucosa, intensifying inflammation.^122,123^ 20–40% of E. coli isolates from CD patients harbor AIEC strains. These strains invade intestinal cells via host cell actin polymerization and microtubule adhesion, thrive, and replicate in macrophages.^124^ Their high replication also triggers the release of substantial TNF-α.^125^

In certain contexts, E. coli strains exhibit protective intestinal effects, notably E. coli Nissle 1917 (EcN), a probiotic that mitigates intestinal inflammation.^126^ Irinotecan-induced gut microbiota disruption and increased intestinal permeability in mice are reversed by EcN treatment, improving dysbiosis and restoring intestinal barrier function. Importantly, EcN also reduced the decrease in transepithelial resistance (TER) and increased FITC-dextran 4000 Da (FD-4) flux caused by the active metabolite SN-38 of irinotecan, and increased the level of transmembrane protein claudin-1, indicating that EcN alleviated the damage to intestinal mucosal monolayer barrier function.^127^ OMV and soluble factors secreted by EcN and E. coli strain ECOR63 can increase the potential of TER, mediate the upregulation of ZO-1 and claudin-14, and downregulate the leakage protein claudin-2 to enhance intestinal epithelial barrier function.^128^ In addition, the ratio of IL-10/IL-12 and IL-10/TNF - α mRNA increased in the explants treated with EcN, and the released OMV strongly upregulated the level of IL-22.^129^

The above results show that Escherichia coli can enhance the immune defense of intestinal epithelium and give intestinal barrier protection, but it may also become a pathogen in some cases. For example, when the number of Escherichia coli in the intestine is too large or mutates, it may cause diseases such as intestinal infection.

### 3.3. Bacteroides fragilis

Non-toxigenic B. fragilis, a probiotic residing in the human gut, has the potential to treat intestinal injury and inflammation by modulating gut microbiota, tight junction protein expression, and inflammatory factors via its metabolites and targeted strains to combat intestinal infections. According to studies, this probiotic maintains the diversity of the gut microbiota and reduces intestinal inflammation by generating functional Foxp3 (+) Treg cells and CD4 (+) T cells that produce interleukin-10 (IL-10) to assist restore the balance between Th-17 and Treg/Th-17.^130–132^ Bf secretes SCFAs, notably butyrate, which suppresses NLRP3-mediated inflammation and macrophage activation. This action mitigates intestinal inflammation and hinders the progression of colitis-associated cancer (CAC).^133^ Specific strains such as Bf FSHCM14E1, NCTC9343 and ZY-312 have shown positive effects on gut health. The inhibition of UC by FSHCM14E1 might be linked to the NF-κB pathway and the release of short chain fatty acids.^134^ The excess level of claudin can induce tight junction fibril formation in fibroblasts cells. The level of claudin-4 was markedly increased in the Cronobacter malonaticus lipopolysaccharides (LPS)-treated group. Claudin-4 was used to characterize the level of epithelial permeability. Pretreatment of B. fragilis NCTC9343 inhibited Cronobacter malonaticus LPS-induced enhancement in mRNA expression of claudin-4, Toll-like receptor 4 (TLR4), and iNOS, in order to lessens intestine structural abnormality, inflammation-induced cytokine infiltration, mucosal and submucosal edema. Furthermore, it improves the Prevotella-9 gut microbiota and reverses the dysbiosis brought on by infection.^135^ Similarly, the Bf strain ZY-312 enhances Muc2 and ZO-1 tight junction protein expression in intestinal epithelial cells, stabilizing Caco-2 monolayer TER while suppressing NLRP3/caspase-1/IL-1β pyroptosis signaling.^136^ It mitigates cell apoptosis, fosters IL-22 and type 3 innate lymphocytes (ILC3) production, and induces STAT3 phosphorylation in the gut mucosa, ameliorating NEC and fostering colonic mucosal rejuvenation.^136,137^ Furthermore, oral ZY-312 administration alleviates diarrhea by boosting commensal A. muciniphila abundance.^138^

However, it is important to mention that there are also toxic strains in Bf, known as Enterotoxigenic B. fragilis (ETBF).^139^ This strain is more abundant in the intestines of patients with IBD such as UC.^140^ ETBF promotes intestinal injury through specific molecular mechanisms. Firstly, ETBF upregulates the expression of histone demethylase JMJD2B through the TLR4-NFAT5 pathway, further removing the inhibitory H3K9me3 marker in the NANOG promoter region to trans activate NANOG, ultimately promoting CRC stemness.^141^ It also induces differentiation of type 17 helper T cells and promotes proliferation of CRC cells by downregulating the expression of miR-149-3p. This process relies on METTL14 mediated N6 methyladenosine methylation.^142^ Secondly, the virulence factor B. fragilis toxin (BFT) secreted by ETBF triggers an inflammatory cascade in colonic epithelial cells. BFT exacerbates inflammation by affecting signaling pathways such as IL-17 R, IL-8, NF-κB, and Stat3.^143,144^ More specifically, BFT triggers IL-8 secretion in colonic epithelial cells by cleaving β-catenin/E-cadherin, leading to NF-κB p65 nuclear translocation. Additionally, BFT activates MAPK pathways independently, compromising intestinal epithelial integrity and amplifying inflammation spread.^145,146^ Lastly, BFT-treated colon cells release MMP-7 and syndecan-2, disrupting the epithelial barrier via AP-1 and ERK activation, further promoting inflammation and tumor progression.^147^

In summary, B. fragilis exhibits both beneficial probiotic properties and potential pathogenic threats to intestinal health. Hence, when investigating and utilizing fragilis pseudomonas, it is imperative to thoroughly evaluate the strain-specific characteristics and associated risks to ensure optimal application for maintaining intestinal well-being.

**Figure 4.**
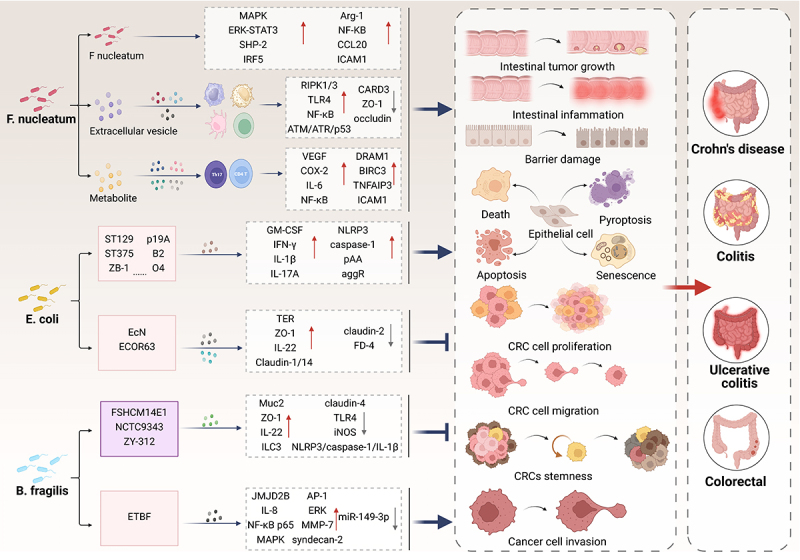
Mechanism of intestinal microflora regulating intestinal injury. F. nucleatum directly invades the intestinal mucosa, generates extracellular vesicles, and activates downstream signaling pathways through metabolites, posing a threat to intestinal health. E. coli and its specific strains can induce the expression of various pro-inflammatory cytokines, and some of its strains have the ability to alleviate intestinal inflammation. B. fragilis fights against intestinal infections by altering the gut microbiota, tight junction protein expression, and inflammatory factors through its metabolites and specific strains. However, its toxic strains can disrupt the integrity of the intestinal epithelial barrier, promoting inflammation and tumor development. These three ultimately affect the occurrence and development of crohn’s disease, colitis, ulcerative colitis, and coloretal.

Absolutely, the specific mechanism of action between gut microbiota and gut health and injury is very complex and multidimensional (Figure 4). By delving into the multifaceted functions and intricate mechanisms of this microbial community and its diverse metabolites, we can unlock innovative strategies and methodologies that effectively prevent and manage gut health concerns.

## 4. The mutual regulation between m6A and gut microbiota

There are interactions between m6A and gut microbiota through multiple pathways, which in turn affect the biological processes of various diseases. A deeper understanding of the crosstalk mechanism between the two will provide some new insights for the management of related diseases.

### 4.1. The effect of m6A on gut microbiota

The role and impact of m6A on gut microbiota is a complex and multidimensional process, involving multiple aspects such as microbial community dynamic balance, gene expression regulation, host metabolism, and immune regulation. Emerging evidence suggests that silencing METTL3 improves aging of nucleus pulposus cells (NPC) and intervertebral disc injury, and restores specific gut microbiota levels in intervertebral disc degeneration (IDD) rats.^148^ METTL14 conditional knockout mice showed a decrease in S24–7 and Lachnospiraceae at week 24, while Bacteroidaceae, Helicobacteraceae, Deferribacteraceae, and Enterobacteriaceae showed a relative increase. In addition, the changes in microbial community structure also affect the expression level of m6A in CD4T cells.^149^ In another study, different characteristics were observed in the fecal fungal communities of YTHDF1 knockout mice and WT mice. In the fecal fungal community of YTHDF1 gene knockout mice, Firmicutes phylum dominates, followed by Bacteroidetes and Verrucomicrobia, while in the feces of WT mice, Proteobacteria and Bacteroidetes are the main phyla. Further analysis showed that the abundance indices of ACE and Chao1 fungal communities in the microbiome of YTHDF1 knockout mice were significantly higher than those in WT mice, indicating an increase in the abundance of Lactobacillus, Bacteroidetes, Lactobacillus, Verrucomicrobiales, and Erysipelotrichales. The WT mouse group mainly clustered Lactobacillales, Eubacteriales, Desulfovibrionales, Bacteroidales, and Chitinophagales.^150^ It is worth noting that subjects carrying FTO risk genotypes have increased gut microbiota diversity, accompanied by high abundance of Blautia bacteria, which is associated with positive improvements in high-density lipoprotein (HDL) and low-density lipoprotein (LDL) levels in subjects.^151^ Besides, 16S rRNA amplicon sequencing revealed that FTO knockout increased the abundance of the Lactobacillus, decreased the abundance of Porphyromonadaceae and Helicobacter, thereby inhibiting gut microbiota inflammation and improving anxiety and depression like behavior.^152^

Apart from exerting a direct influence on the composition of gut microbiota, host genetics may also indirectly regulate the composition and function of gut microbiota by affecting gene expression in host cells.^153,154^ Vitamin D receptor (VDR) plays a pivotal role in maintaining intestinal homeostasis and safeguarding hosts against bacterial infiltration and infection, significantly impacting the gut microbiota composition. Mice deficient in VDR exhibit marked alterations in β diversity and specific bacterial taxa, notably Parabacteroides.^155^ Furthermore, ablation of VDR in mice has been observed to facilitate severe Salmonella invasion within the gut, resulting in an elevated bacterial burden and mortality rate.^156^ It is noteworthy that the translation efficiency of VDR can be improved by knocking down YTHDF1, while depletion of METTL14 will reduce the expression of VDR mRNA.^157,158^ This indicates that the expression of VDR is regulated by a multitude of m6A regulatory factors, but its specific regulatory mechanism needs to be elaborated. Cytokines similar to VDR also include MUC family and suppressor of cytokine signaling (SOCS). For instance, the transmembrane MUC1 within the mucin family facilitates bacterial invasion through its interaction with β1-integrin.^159^ Intriguingly, ALKBH5 diminishes the m6A modification on MUC1 mRNA, destabilizing the latter and subsequently reducing MUC1 expression.^160^ Conversely, the absence of METTL3 eliminates m6A modification on SOCS2 mRNA, thereby enhancing SOCS2 RNA expression. Furthermore, the m6A-mediated degradation of SOCS2 mRNA relies on the YTHDF2-dependent pathway.^161^ In immature T cells lacking METTL3, the m6A markers on SOCS family genes, including SOCS1, SOCS2, and Cish, exhibit slower mRNA degradation rates, leading to elevated mRNA and protein expression levels.^162^ These findings highlight the pivotal role of m6A regulatory factors in modulating the expression of cytokines such as VDR, MUC, and SOCS, thereby influencing the delicate balance of the gut microbiome. This also implies that gut microbiota may reciprocally impact the m6A modification patterns in host cells, underscoring the intricate interplay between the gut ecosystem and host cellular processes.

The above indicates that m6A modification may affect the gut microbiota in two ways, one through direct regulation, and the other through indirect regulation of gut microbiota homeostasis by affecting gene levels closely related to the gut microbiota. However, more investigation and identification are required to completely comprehend the molecular process of how host m6A alteration accurately manipulates intestine microbial populations.

### 4.2. The effect of gut microbiota on m6A

The intestinal microbial community exerts a profound influence on the host’s apparent transcriptomic profile, with its metabolites, notably folic acid and choline, functioning as crucial methylation donors that facilitate the smooth progression of DNA methylation within the host.^163^ In a germ-free (GF) mouse model, a significant increase in overall DNA methylation levels was observed in epithelial cells, while methylation levels in small intestine segments (duodenum, jejunum, and ileum) remained relatively stable, which may be closely related to the decrease in DNA methyltransferase activity and folate cycling concentration.^164^ The microbiome’s existence exerts a tissue-specific influence on the levels of m6A within cellular mRNA. M6A-MERIP sequencing analysis unveiled pronounced disparities in m6A modification patterns between specific pathogen free (SPF) and GF mice across intestinal, hepatic, and cerebral tissues, with the brain tissue exhibiting heightened sensitivity to these alterations. In the brain tissue of GF mice, there was a marked upregulation of both m6A methylating enzymes (METTL3 and METTL14) and demethylating enzymes (ALKBH5 and FTO), forming a stark contrast to SPF mice.^165^ Certain specific microorganisms directly affect the m6A pattern of the host. For example, Saccharomyces boulardii upregulates METTL3 levels through an m6A dependent mechanism, which helps restore metabolic homeostasis in the gut microbiota.^166^ Infection with intestinal pathogenic bacteria such as enterotoxin Escherichia coli K88 (E. coli K88) promotes RNA m6A methylation in intestinal epithelial cells, induces the interaction between transcription factors FOXO6 and METTL3, activates GPR161 transcription, and upregulates the expression of intestinal β-defense factors.^167^ Heat-killed Salmonella typhimurium (HKST) infection significantly increased m6A modified mRNA levels and WTAP expression in THP-1 cells.^168^ It is noteworthy to mention that antibiotic induced intestinal dysbiosis can trigger a series of gene expression changes, including downregulation of YTHDC1, FTO, and methyl donor related proteins (METTL16, MAT1A and MAT2A). At the same time, metabolites such as bile acids also downregulate METTL3 and METTL14, leading to remodeling of the host transcriptome and m6A exon.^169^ Furthermore, the utilization of nonstarch polysaccharides (NSPs) by gut bacteria not only reshapes the microbial community structure, but also exerts the m6A RNA methylation process by modulating the supply of methyl donors, ultimately improving cancer.^24^

The gut microbiota may potentially modify host m6A modifications via intermediate mediators such as microbial metabolites and a cascade of signaling pathways. BAs are signaling molecules with metabolic functions that play important roles in cellular processes. Deoxycholic acid (DCA), as one of the most common BAs, regulates the expression of downstream factors through m6A dependent post transcriptional modification by promoting the dissociation of the METTL3-METTL14-WTAP complex, ultimately exerting tumor suppressive effects.^170^ Vitamin B12, as a regulator of gut microbiota ecology, its deficiency leads to a decrease in SAM levels and m6A levels in mRNAs.^171^ Analogously, butyric acid secreted by intestinal flora can downregulate METTL3, leading to a diminished methylation level of FOSL2 m6A, ultimately reducing downstream NLRP3 protein and inflammatory cytokine IL-6 and TNF-α expression, thereby alleviating inflammatory responses.^172^ Previous investigations have hypothesized that the AMPK/SIRT1 and IL-6/STAT3 pathway appear to be crucial intermediaries of microbiota regulation of host m6A.^173^ For example, Lactobacillus sakei upregulates the AMPK/SIRT1 pathway in mice, and SIRT1 promotes FTO degradation through RANBP2 mediated FTO-SUMOylation, further altering m6A methyl groups.^174,175^ Diverse bacteria, such as Escherichia coli, Staphylococcus aureus, and Gram negative bacteria, can activate IL-6 and STAT3. This activation, in turn, augments the expression of the m6A methyltransferase complex (METTL3/METTL14/WTAP), enhancing its mRNA stability and translation in an m6A-IGF2BP2-dependent fashion.^176^ Additionally, gut microbiota may potentially modulate host M6A modifications through the induction of reactive oxygen species (ROS) and LPS, but this still needs further exploration.^177^

**Figure 5.**
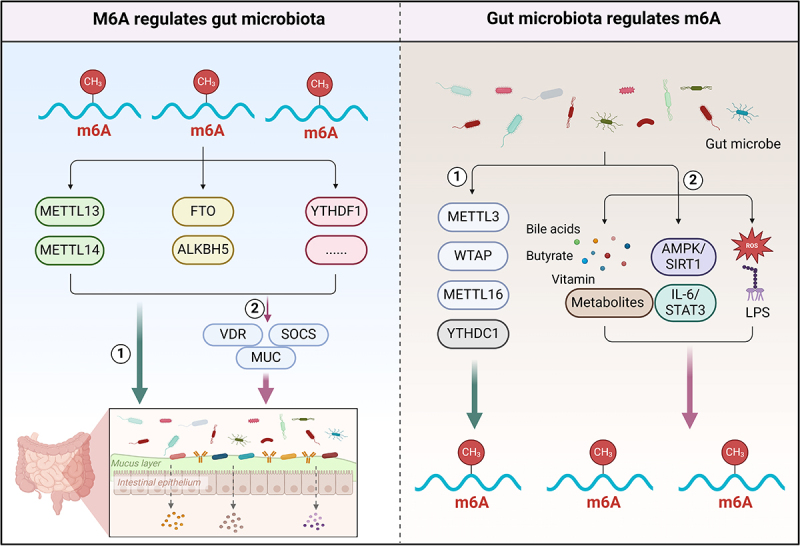
Interaction between m6A and gut microbiota. M6A modification can not only directly regulate gut microbiota, but also indirectly regulate gut microbiota homeostasis by affecting gene levels closely related to gut microbiota (VDR, SOCS, and MUC). The gut microbiota can be directly modified by affecting M6A, and can also be influenced by its metabolites, a series of signaling pathways (AMPK/SIRT1, IL-6/STAT3, LPS, and ROS), and other intermediate mediators.

In summary, via carefully controlling the m6A RNA modification profile in the host’s epigenetic transcriptome, the gut microbiota, either as a whole or as a particular member, plays a critical role in sustaining host health, disease progression, and intervention treatment. Nevertheless, further research is still needed to determine the precise biochemical mechanism behind the relationship between microbiota and m6A methylation (Figure 5).

## 5. M6A and gut microbiota in gut injury and health

The gut microbiota and its diverse metabolites exert a profound influence on the m6A modification levels within the host’s intestinal environment. Folic acid synthesized by Lactobacillus and Bifidobacterium can be used to synthesize the methyl donor S-adenosylmethionine (SAM) via the methionine cycle, enhancing DNA methylation and mRNA m6A in the intestine. In addition, vitamins B2, B6, and B12 sourced from the microbiota also participate in the one carbon cycle to synthesize SAM and regulate methylation modifications, thereby maintaining healthy intestinal development.^23^ The m6A modification spectrum in cecal tissue is also controlled by the gut microbiota. The m6A peak in cecal transcripts of conventional (CONV) mice and GF sterile mice shows varying degrees of methylation. Two types of bacteria, Akkermansia muciniphilia (A. muciniphilia) and Lactobacillus plantarum (L. plantarum), affect host m6A modification in the cecum and liver, with L. plantarum having a stronger effect on host RNA methylation. In the absence of gut microbiota, GF mice display significantly reduced mRNA levels of the methyltransferase METTL16 in the small intestine, colon, and cecum, whereas METTL3 mRNA levels in the small intestine are slightly elevated. The protein level of Mat2a in the cecum of GF mice decreased and was hypomethylated.^178^ Mat2a, as an enzyme that generates the methyl donor SAM required for methyltransferase activity, is associated with decreased expression and altered methylation profiles.^179^ This underscores the significance of transcriptome modifications in the intricate relationship between gut microbiota and their hosts. Fecal microbiota transplantation (FMT) utilizing fecal triglycerides has the potential to mitigate obesity and lipid metabolism disorders induced by a high-fat diet (HFD), while simultaneously modifying the composition of the jejunal microbiota. This beneficial effect is intimately tied to the modulation of m6A modification levels within the jejunum and the expression patterns of m6A writer and eraser genes. The mRNA level of YTHDF3 was detected to decrease under HFD treatment. The high concentration of wild boar feces suspension prevented the decrease in m6A levels induced by HFD. Lactobacillus and Romboutsia are dominant bacteria in the jejunum, and their abundance can be regulated by FMT, resulting in higher levels of m6A and subcutaneous fat weight, as well as lower levels of triglycerides in the jejunum.^180^ This finding suggests that FMT exerts its regulatory effect on lipid metabolism by altering m6A modification levels in the jejunum through modulation of the abundance of Lactobacillus and Romboutsia. An additional study corroborates the finding that mice subjected to a HFD exhibited reduced levels of m6A and m5C modifications in their small intestinal RNA compared to control mice, and the expression of “writers” such as METTL3, METTL14, and WTAP is downregulated. There were significant differences in 16 bacterial genera between the two groups of mice, among which Lactobacillus and Eubacterium-xylanophyllum demonstrating a pronounced positive correlation with m6A levels. Furthermore, the fecal microbiota of obese mice was transplanted into antibiotic treated mice, and the results showed that the dysbiosis of the microbiota in diet induced obesity led to a decrease in the intestinal expression of m6A levels written into genes (METTL3, METTL14 and WTAP). This indicates that m6A levels were first reduced through HFD, and then further reduced through intervention of the fecal microbiota in HFD mice.^181^ This also reflects the bridging role of the microbiome in different dietary patterns involved in m6A methylation modification.

Research has demonstrated that specific bacteria can impact gut health by inhibiting the augmentation of m6A, and correspondingly, alterations in m6A levels can modulate the constitution of gut microbiota, thereby influencing the progression of various diseases. The accumulation of F. nucleatum in the intestine has been linked to intestinal barrier dysfunction,^94,182^ and it selectively attracts tumor-infiltrating immune cells, fostering a proinflammatory milieu that fosters CRC progression.^100,110^ Notably, F. nucleatum was found to significantly diminish m6A modification in CRC cells and patient-derived xenograft tissues (PDX), accelerating CRC. Importantly, FOXD3, a crucial transcription factor for the m6A methyltransferase METTL3, is inhibited by F. nucleatum. This occurs through suppression of the YAP signaling pathway, activated by the Hippo pathway in CRC cells, ultimately decreasing METTL3 transcription. Consequently, reduced METTL3 expression leads to decreased m6A levels, specifically affecting kinesin family member 26B (KIF26B) mRNA. This, in turn, reduces YTHDF2-mediated degradation of KIF26B mRNA, upregulating KIF26B expression and contributing to F. nucleatum -induced CRC cell invasion and metastasis.^183^ Butyrate, a metabolic byproduct of intestinal flora, exerts its anti-CRC effects by diminishing m6A levels and suppressing the expression of METTL3 in CRC cells. This suppression disrupts the regulatory mechanism whereby METTL3 modulates the expression of cyclin E1 (CCNE1) by methylating the m6A site within its 3’-UTR in CCNE1 mRNA, thereby inhibiting CRC progression. Notably, overexpression of METTL3 can counteract the growth-inhibitory and colony-formation-suppressing effects of butyrate treatment on CRC cells.^184^ These findings suggest that butyrate downregulates CCNE1 expression in a manner that is dependent on the m6A-METTL3 axis. Furthermore, exposure to ETBF disrupts the levels of primary microRNA-149-3p (pri-miR-149-3p) and represses the expression of METTL14. This downregulation of METTL14 subsequently impairs the processing of pri-miR-149, resulting in decreased levels of mature miR-149-3p, and finally promotes the proliferation of CRC cells.^142^ On the contrary, A. muciniphilia was significantly enriched in the fecal fungal community of YTHDF1 knockout mice, while A. muciniphilia can affect specific m6A modifications in mice.^150,178^ Knockout of YTHDF1 reduces the modification ability of m6A, hinders mRNA translation, and affects downstream biological functions.^185^ Therefore, we speculate that the absence of YTHDF1 may be due to an increase in A. muciniphilia cell colonies weaken the ability of RNA binding proteins to recognize m6A, ultimately delaying disease progression. However, further research is needed to explore the exact mechanism of gut microbiota and mRNA methylation levels in diseases

**Figure 6.**
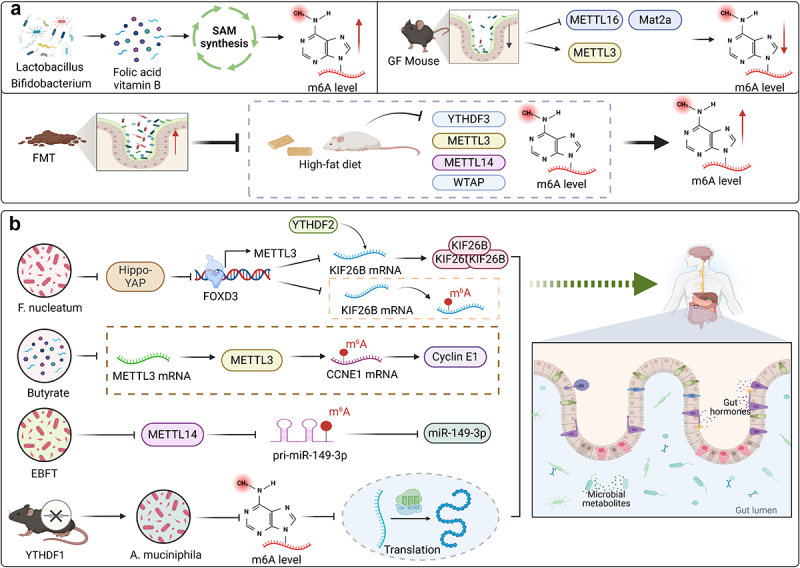
Interaction between m6A and intestinal microflora in intestinal injury and disease. (a) Folic acid synthesized by gut microbiota can be converted into the methyl donor SAM through the methionine cycle, increasing mRNA m6A in the gut. When the gut microbiota is deficient, M6A in the cecum of GF mice is hypomethylated. FMT can prevent obesity and lipid metabolism disorders induced by HFD, and prevent the decrease in m6A levels induced by HFD. (b) F. nucleatum, Butyrate, ETBF, and A. muciniphila may affect intestinal tissue and the progression of intestinal related diseases by altering the host’s RNA m6A spectrum.

The above studies underscore that dynamic epigenetic modifications of intestinal tissue strongly mediate the crosstalk between gut microbiota and intestinal homeostasis. Gut microbiota may alter the host’s RNA m6A spectrum, which may further affect intestinal tissue and the development of intestinal related diseases. However, further research is needed to explore the individual and combined effects of gut microbiota and host RNA m6A modification, which can provide new insights into gut diseases and develop new strategies for the diagnosis and treatment of gut disease patients (Figure 6).

## 6. Conclusion and prospect

m6A, the most prevalent and reversible entity within the intricate landscape of RNA modifications, assumes a pivotal role in meticulously orchestrating gene expression patterns, safeguarding mRNA stability, and meticulously modulating splicing events.^186,187^ It is worth noting that there is an intricate interplay between subtle fluctuations in m6A modification levels and the emergence as well as the progression of various intestinal diseases. In particular, during the pathological trajectory of inflammatory bowel disease, m6A modifications actively engage in the disease process, exerting multi-level regulatory effects on gene expression profiles, cell differentiation pathways, and immune response networks.^38,188^ The gut microbiota, a vital component of the host’s microenvironment, not only plays a pivotal role in preserving physiological balance and immune homeostasis but also engages in intricate interactions with host cells via its metabolites, significantly influencing the m6A modification status of host RNA.^173^

In recent years, the intersection of gut microbiota and intestinal diseases via the epigenetic regulatory mechanism of m6A modification has emerged as a burgeoning frontier in scientific research. While advancements have been made in this realm, the complex molecular mechanisms underpinning this interplay remain largely uncharted territories. Consequently, future endeavors should prioritize delving into the specific molecular pathways that regulate the interaction between gut microbiota and host RNA m6A modification. Additionally, the development of m6A-based detection methodologies for early diagnosis and prognostic assessment of intestinal diseases represents a promising direction. Attention should also be paid to whether the microbial regulation of m6A can reverse the microbial population through host immune factors (such as IL-10 and TNF-α), and whether there is negative feedback to maintain microbe-host homeostasis. Concurrently, the pursuit of targeted drugs and therapeutic strategies aimed at m6A modification holds the potential to revolutionize the treatment landscape for intestinal diseases, offering robust scientific foundations for enhancing patient prognosis and quality of life.

## Data Availability

The data that support the findings of this study are available from the corresponding author, [XAZ], upon reasonable request. https://orcid.org/0000-0002-7854-4134.
